# Supplementary material for: Mechanistic Modelling of Slow and Fast NHEJ DNA Repair Pathways Following Radiation for G0/G1 Normal Tissue Cells
Source: Cancers (Basel). 2021 May 3;13(9):2202. doi: 10.3390/cancers13092202 (PMC8124137; doi:10.3390/cancers13092202)
Supplement: Supplementary file 1 [file cancers-13-02202-s001.zip › cancers-1190122-supplementary.pdf]

2Gy Photon

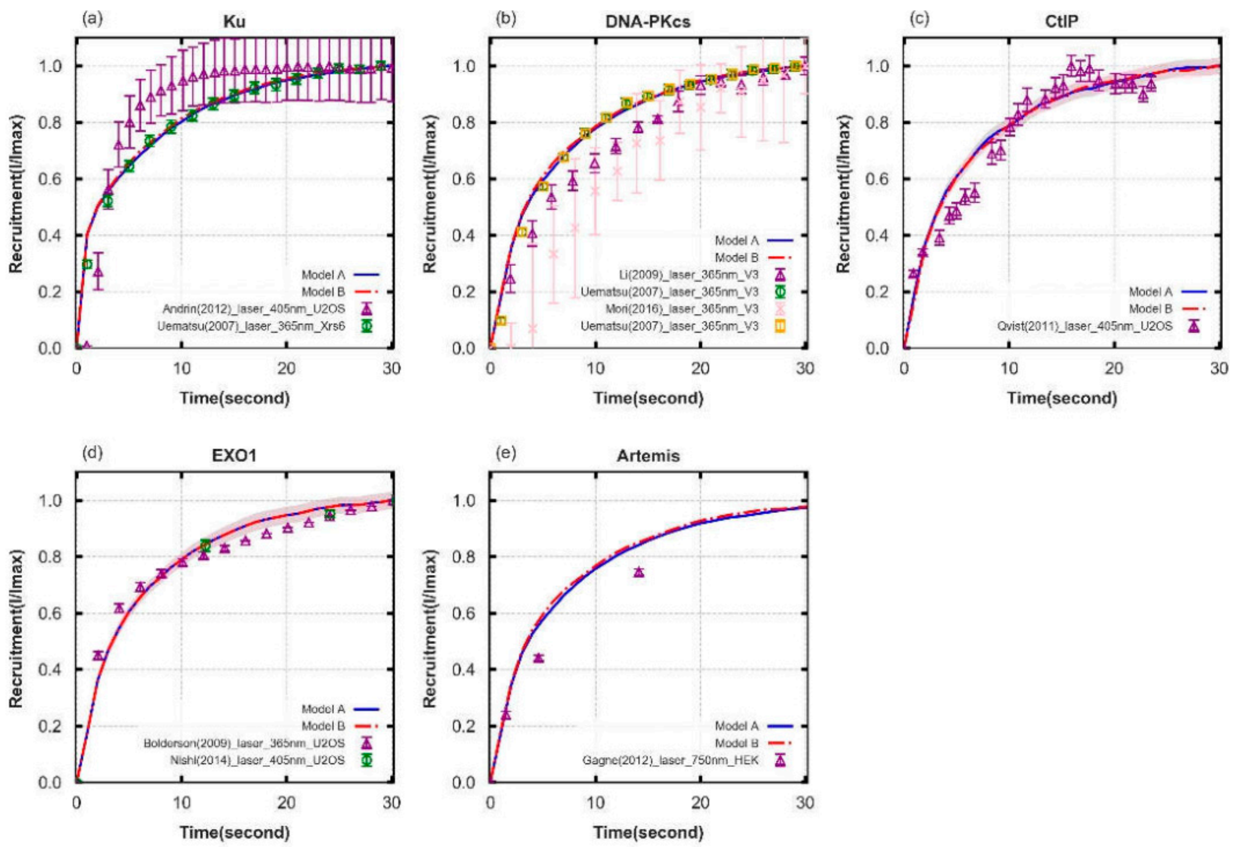

1.3Gy Photon

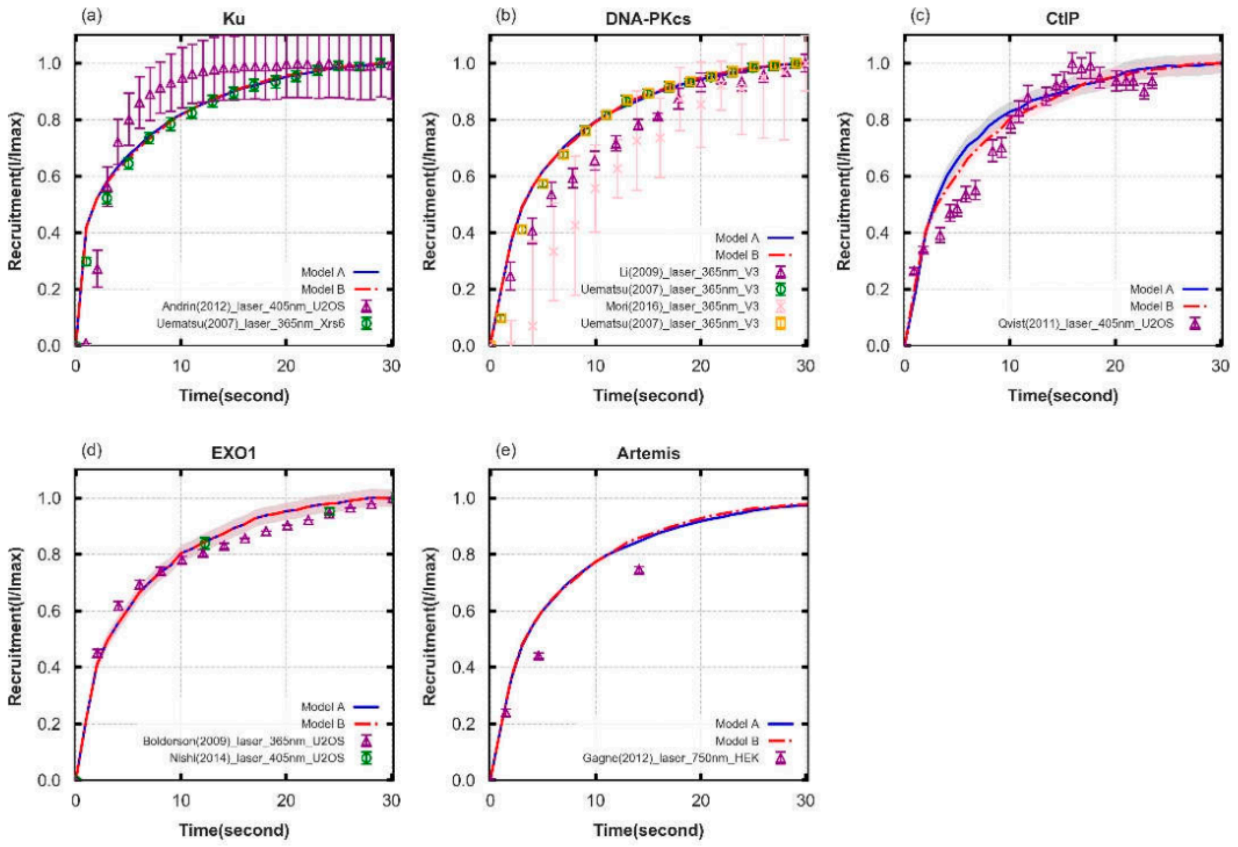

0.5Gy Photon

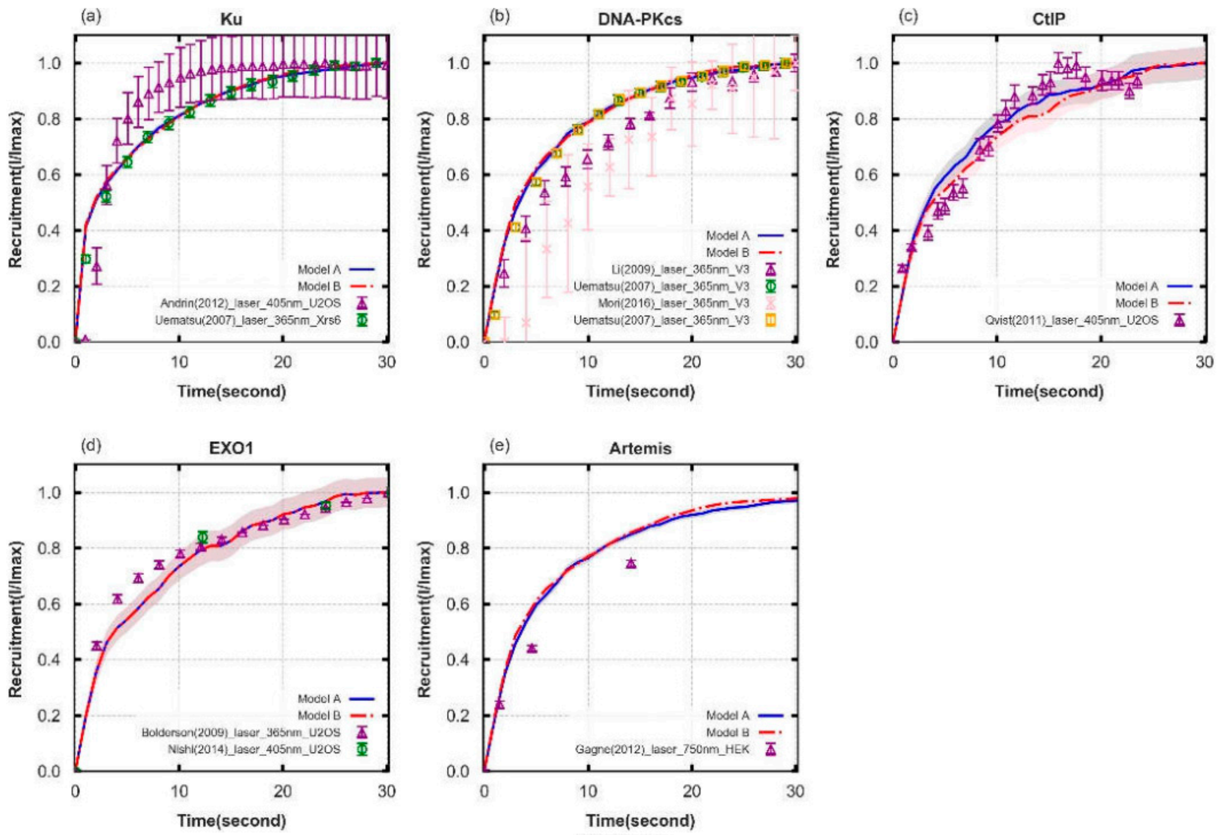

4Gy Photon

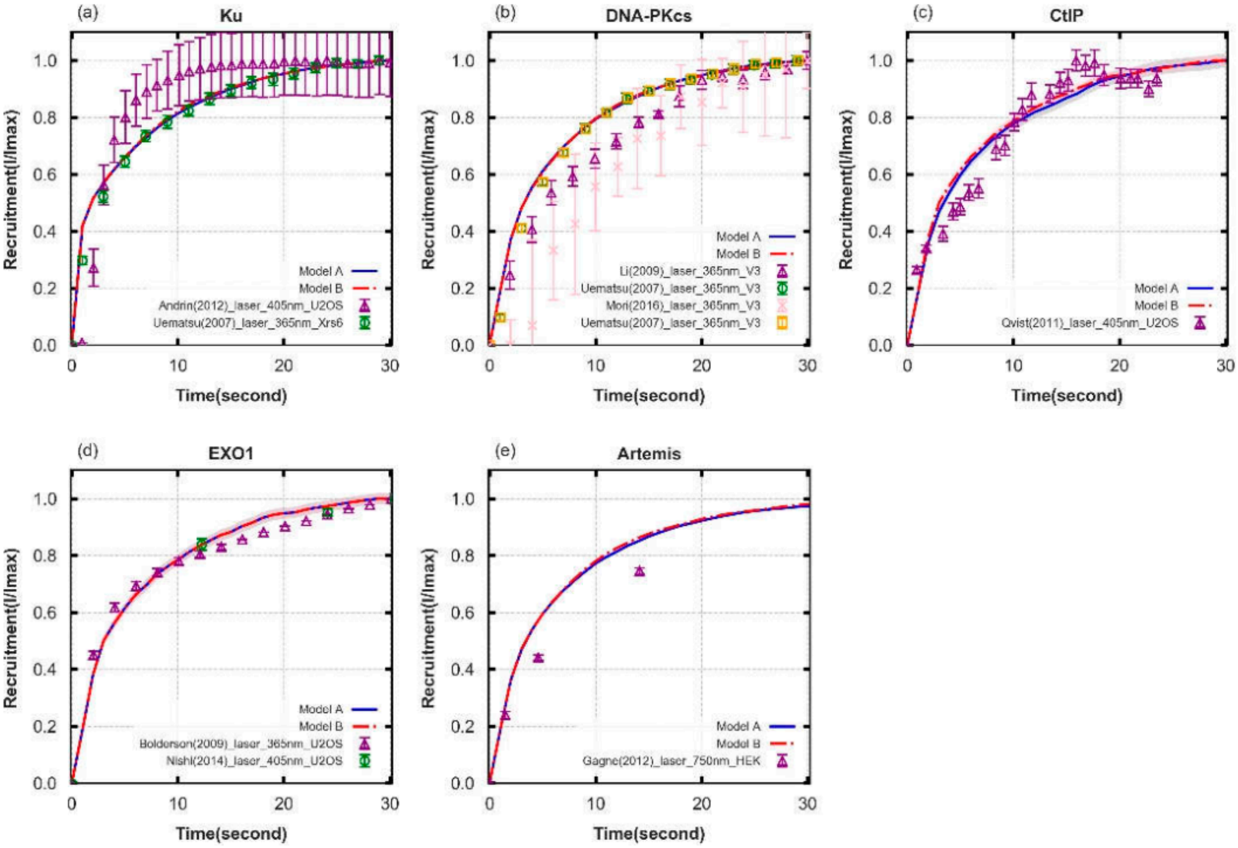

2.0 GyProton

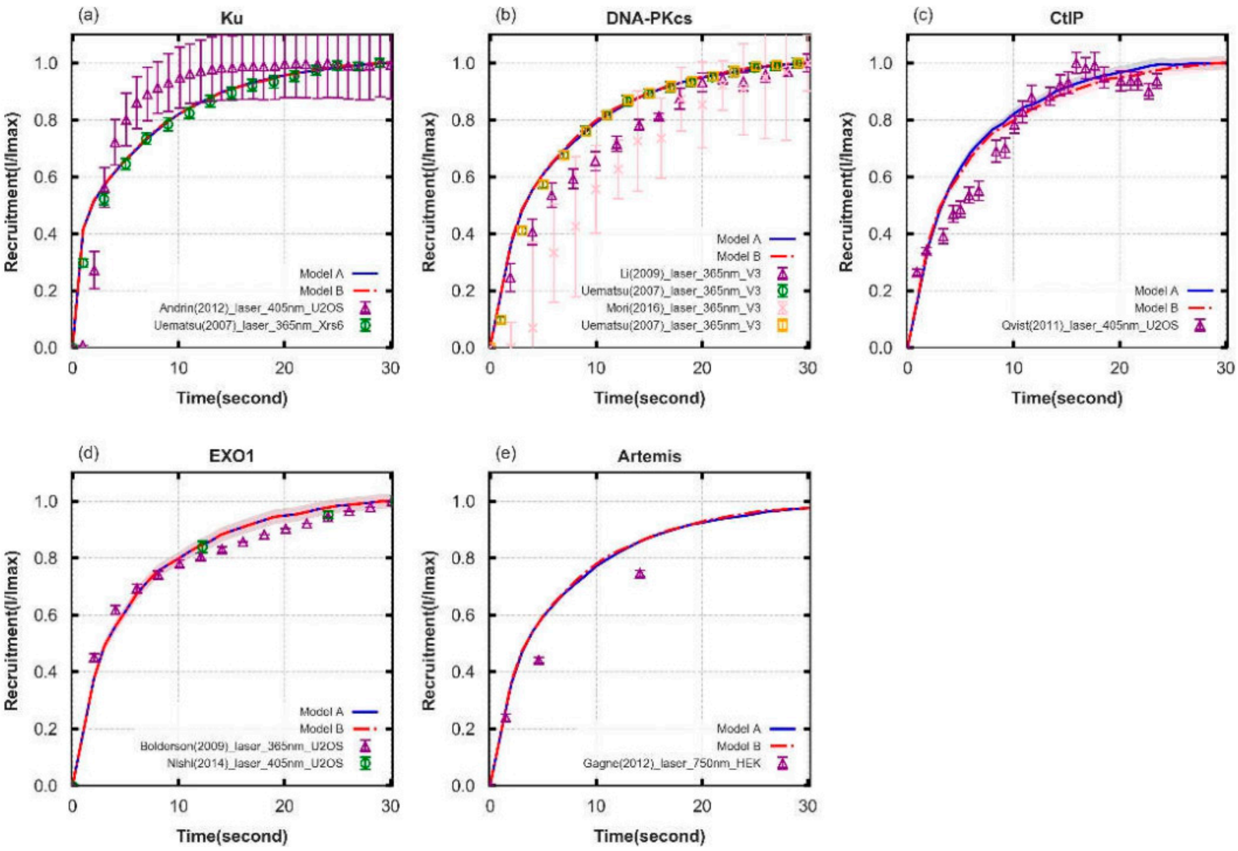

0.5Gy Proton

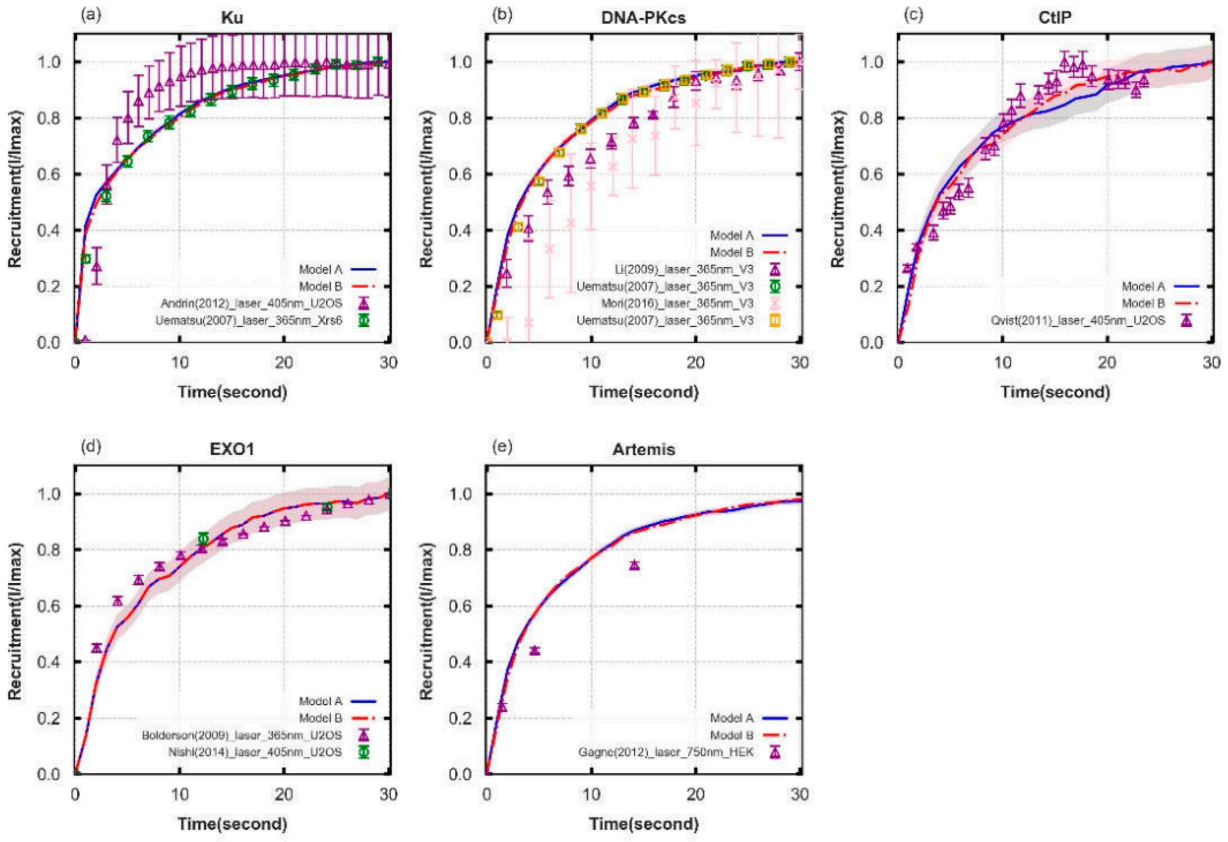

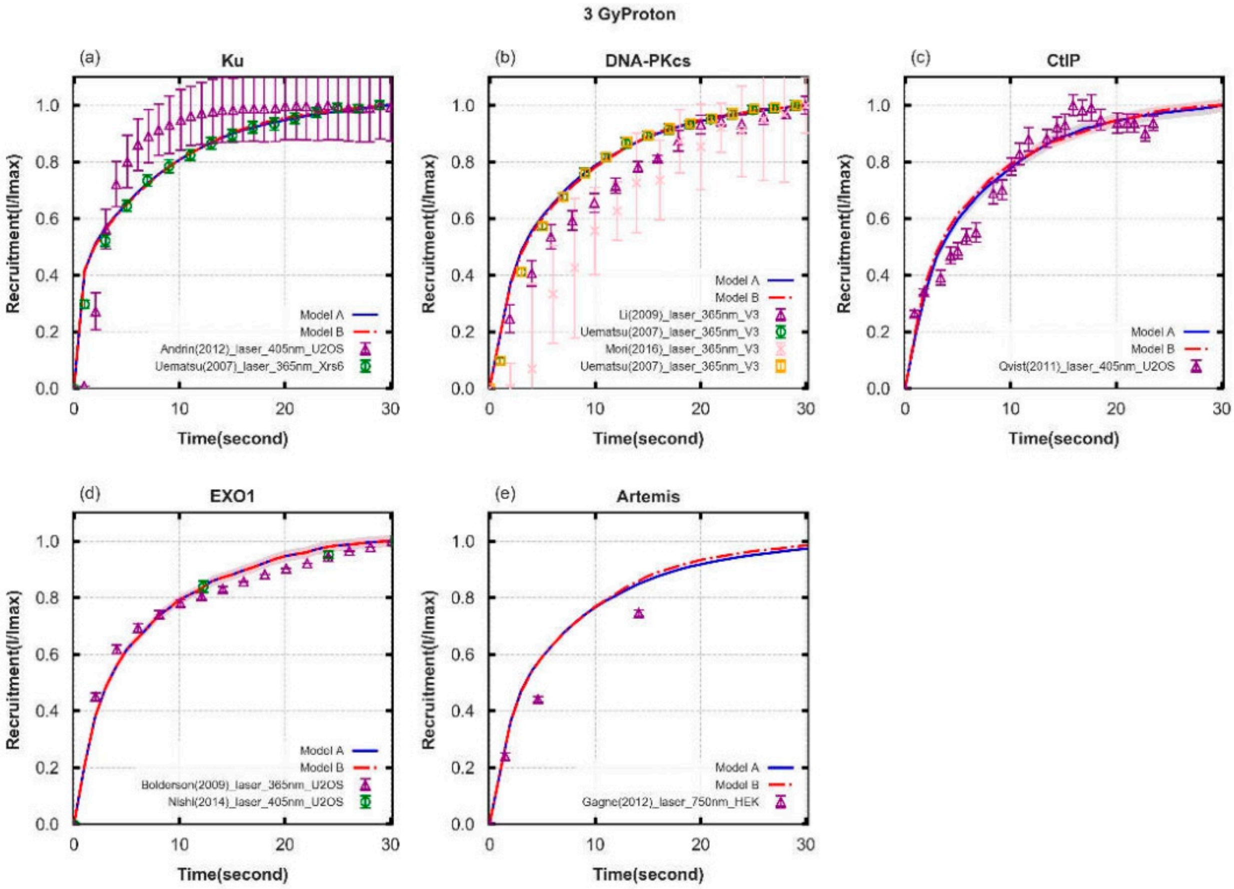

Figure S1. Comparison of the recruitment kinetics of 8 types of irradiationapplied on the normal tissue cellssimulated in Models A, and B.Figure A1(a)-(d)are results for photons with 0.5, 1.3, 2and 4Gy doses; (e)-(h) are results for protons with 0.5, 2and3Gydoses.

1.3GyPhoton Artemi-KO

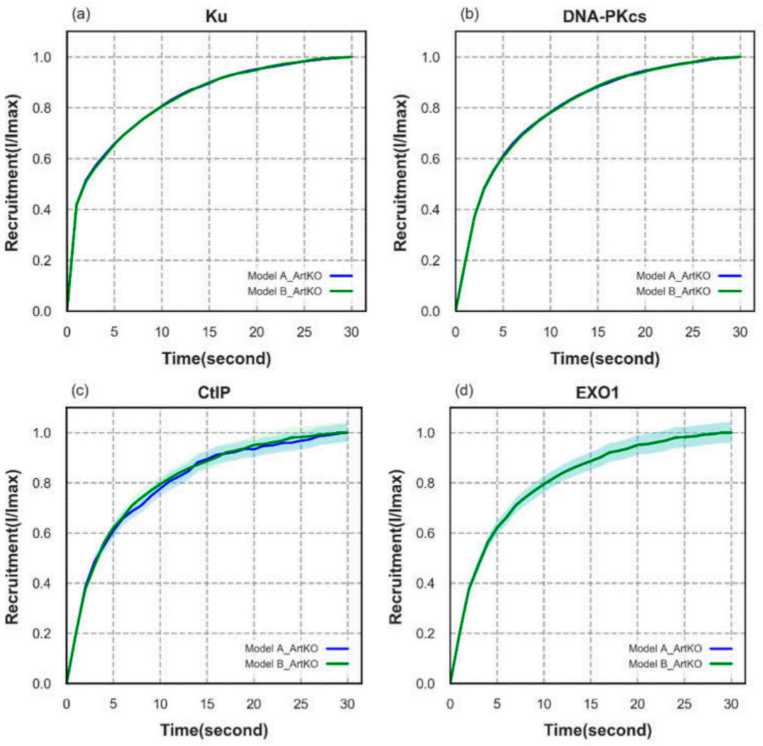

2GyPhoton Artemi-KO

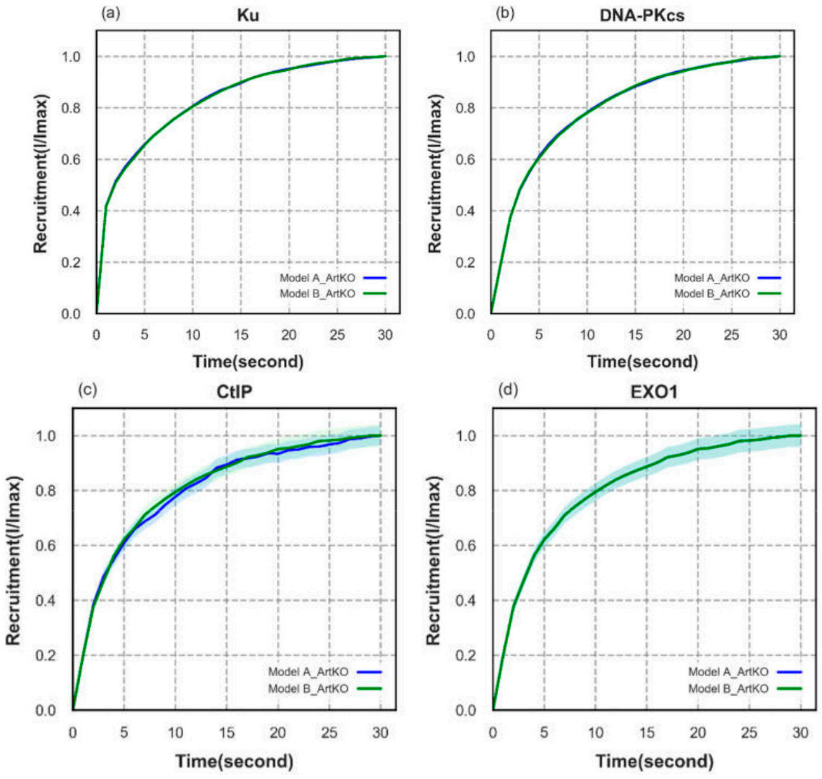

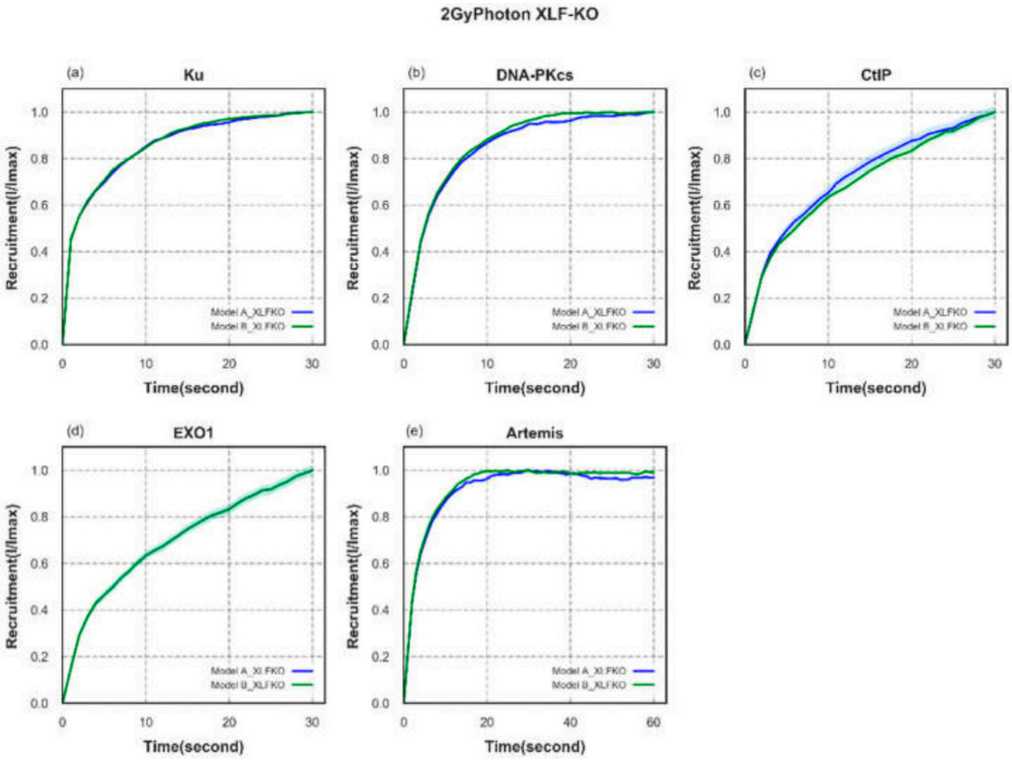

Figure S2. The recruitment kinetics of Arteims-deficient and XLF-deficient cell system corresponding to the repair kinetics in Figure 7.

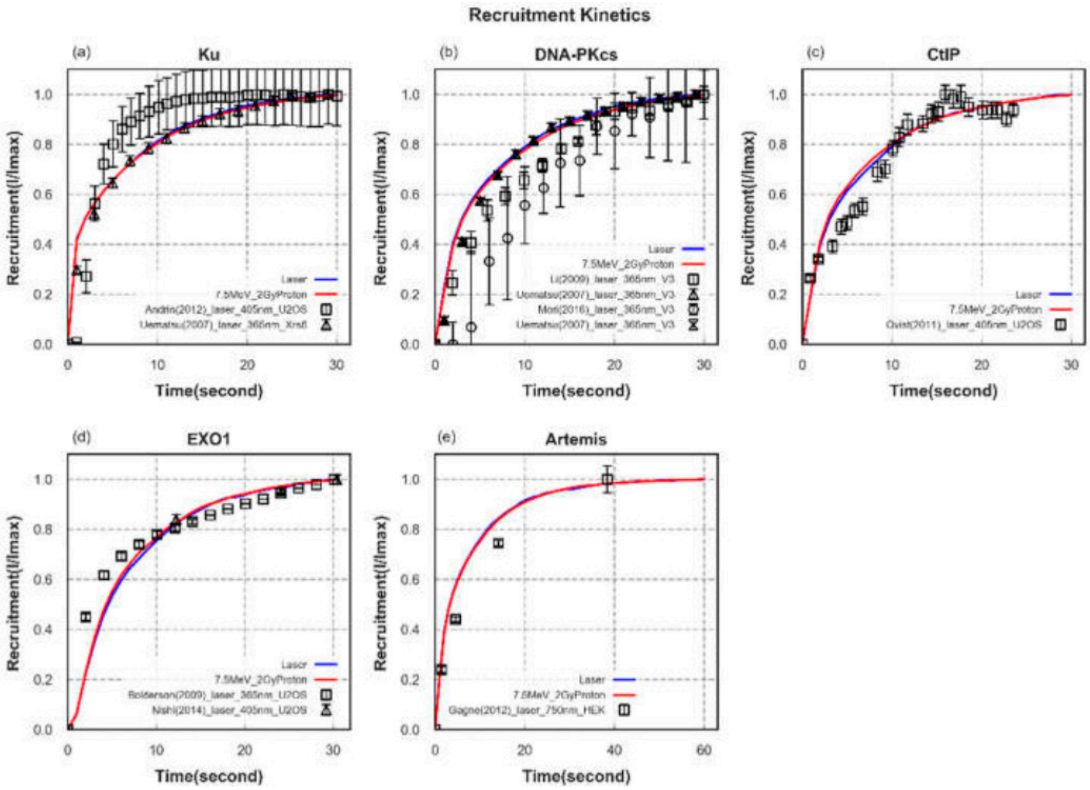

Figure S3. Recruitment kinetics of proteins when irradiated with laser and 4Gy proton(7.5MeV).Each DSB is scored as occurring within heterochromatin or euchromatin. The exact proportion of the genome comprising of either form of chromatin compaction varies within the literature.

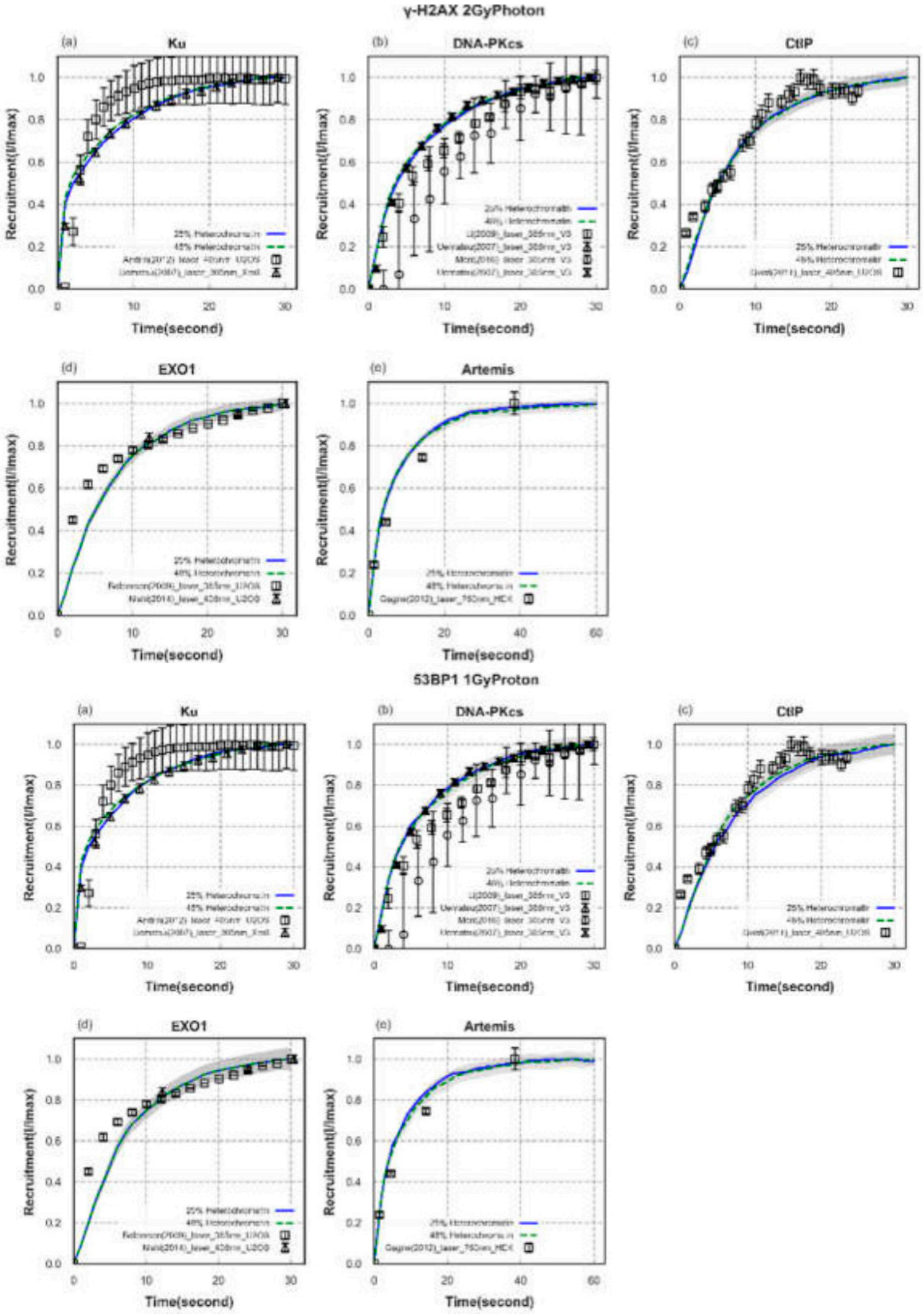

Figure S4.The recruitment kinetics of “Parallel” pathway assigned with 25% and 48% heterochromatin (forced to recruit DNA-PKcs only, and euchromatin is forced to recruit CtIP) under 2Gy photon/1Gy 1.7keV/um proton irradiation,corresponding to the repair kinetics in Figure 6.

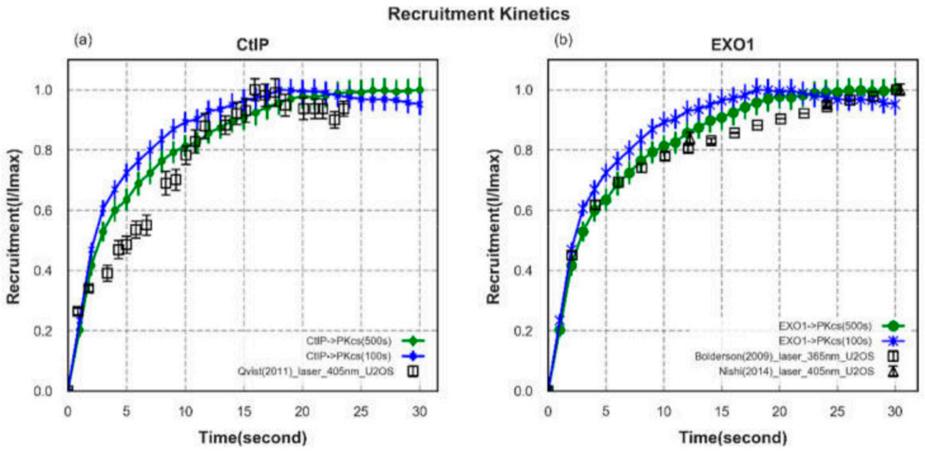

Figure S5.The recruitmentkinetics of CtIP and EXO1 with 2 different time components when loading Artemis:PKcscomparing with experimentaldata.

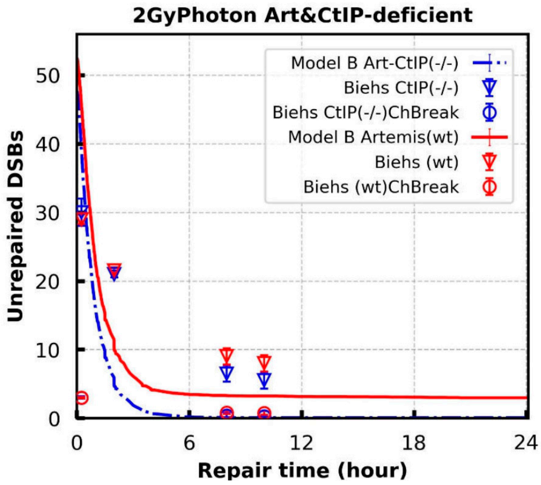

Figure S6.Comparison of unrepaired DSBs in Model B (lines) with the number of γ-H2AX foci obtained from experiments (symbols)for Artemis&CtIP -deficient and wild-type cell lines exposed to 2 Gy X-rays.

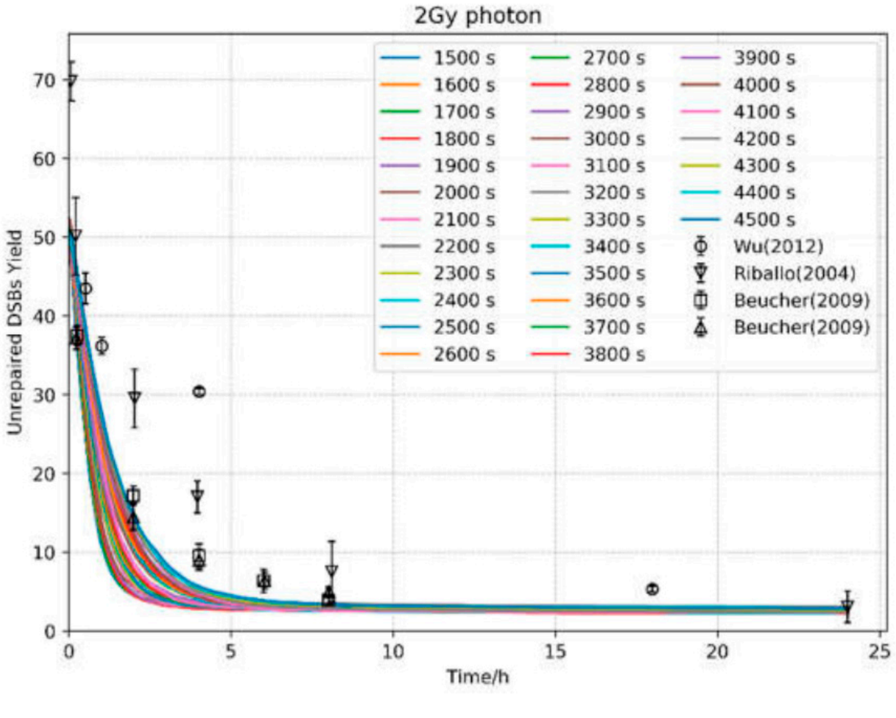

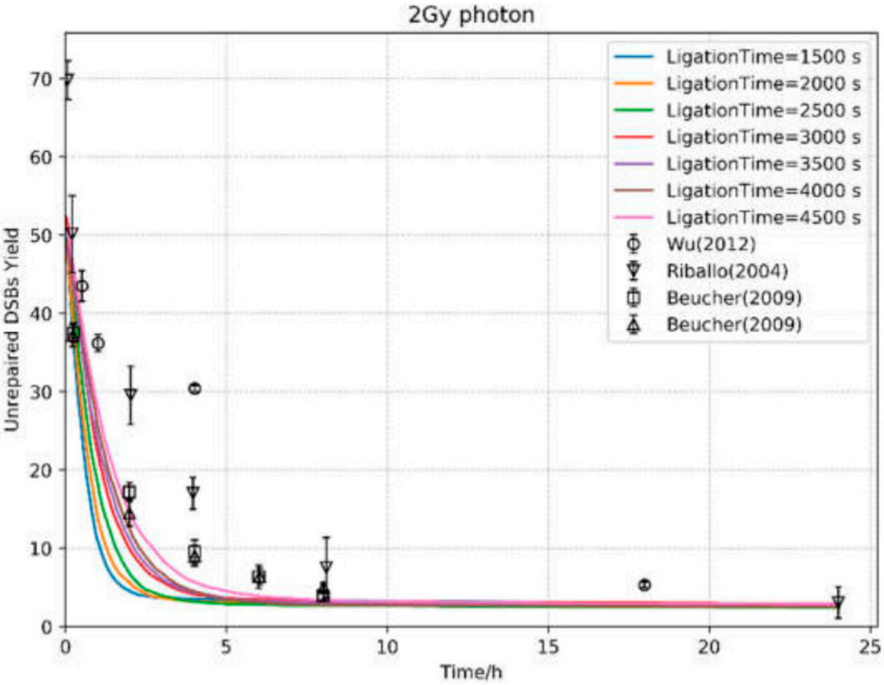

Figure S7.Comparison of unrepaired DSBs in Model B with different ligation time in steps of 100s (from 1500 up to 4500s) exposed to 2 Gy X-rays.

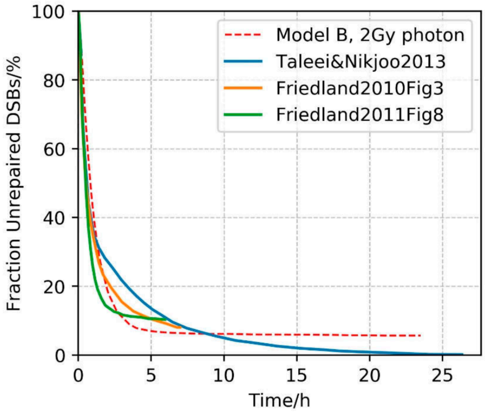

Figure S8.Comparison against three other models of NHEJ published in literature. e include here comparison against three other models of NHEJ published in literature (Nikjoo et al, 2013,http://dx.doi.org/10.1016/j.mrgentox.2013.06.004; Friedland et al, 2011, doi:10.1093/rpd/ncq383; Friedland et al, 2011. doi:10.1016/j.mrfmmm.2011.01.003). The model by Friedland et al. focuses on developing a model of c-NHEJ, which, in light of the work done by the Jeggo group, we would now classify as resection-independent. The model by Nikjoo et al. expands this by additionally including the mechanisms of MMEJ and chromatinrelaxation. The aim of this research was to develop a model that incorporates the specific mechanisms of resection-dependent NHEJ along with resection-independent NHEJ in G0/G1, whilst maintaining an acceptable fit to literature data. Here we see agreement between model B and other published models.

Table S1. Summary of goodness-of-fit metrics for recruitment kinetics between "Parallel" pathway (A) and "Entwined" pathway (B) and experimental data from literature.

Protein	Publication	DF	χ^2/DF*	
			A	B
Ku70/80	Andrin *et al. fig.8* [69]	28	0.046	0.042
	Uematsu *et al. fig.2c* [53]	14	4.068	4.426
DNA-PKcs	Li *et al. fig.2* [70]	14	10.980	12.266
	Uematsu *et al. fig.2* [53]	14	5.439	6.836
	Mori *et al. fig.3* [71]	14	2.295	2.430
	Uematsu *et al. fig.1d* [53]	14	5.272	6.548
CtIP	Qvist *et al. fig.5d* [42]	23	6.452	7.423
EXO1	Bolderson *et al. fig.1b* [72]	12	6.28e8	1.29e9
	Nishi *et al. fig.6g* [41]	2	0.579	0.728
Artemis	Gagne *et al.* [43]	4	11.962	13.778
Overall Statistics of Sum (min)			21.334	24.401
Overall Statistics of Sum (max)			6.28e8	1.29e9

* DF equals data points to compare minus the number of model fitting parameters.

Table S2. Summary of goodness-of-fit metrics metrics (χ2/DF) for repair kinetics between Models A, B, and experimentaldata setsfrom foci data.

Experiments			DF	Simulations	
				Chi-square/DF	
γ-H2AX for normal cell lines				A	B
4 Gy proton/7.5 MeV	NHLF	Fig 3c. from Wu *et al* [49]	3	637.080	742.569
3 Gy proton/32 MeV	MEF	Fig 2c. from Oeck *et al* [73]	4	11.040	8.451
3 Gy proton/187 MeV	MEF	Fig 2c from Oeck *et al* [73]	4	98.701	9.653
2 Gy proton/7.5 MeV	NHLF	Fig 3b. from Wu *et al* [49]	3	27.349	45.649
0.5 Gy proton/7.5 MeV	NHLF	Fig 3a. from Wu *et al* [49]	3	17.739	57.578
4 Gy photon	NHLF	Fig 3c. from Wu *et al* [49]	3	444.767	35.572
2 Gy photon	NHLF	Fig 3b. from Wu *et al* [49]	3	254.912	520.904
2 Gy photon	MCR-5	Fig 1b. from Riballo *et al* [32]	5	10.042	8.700
2 Gy photon	C2906	Fig 2a. from Beucher *et al* [50]	4	69.384	7.331
2 Gy photon	MEF	Fig 2b. from Beucher *et al* [50]	4	48.538	6.285
1.3 Gy photon	1BR3	Fig 2a. from Beucher *et al* [50]	3	32.335	23.886
0.5Gy photon	NHLF	Fig 3a. from Wu *et al* [49]	3	21.296	25.647
0.5Gy photon	MEF	Fig.5a from Ahmed *et al* [44]	4	2.382	2.181
0.5Gy photon	Sertoli	Fig.2b from Ahmed *et al* [44]	3	9.612	13.876
Average				118.780	107.734
γ-H2AX for Artemis-deficient cell lines				A	B
2 Gy photon	MEF	Fig 2b. from Beucher *et al* [50]	4	9.910	15.389
2 Gy photon	CJ179	Fig 2a. from Beucher *et al* [50]	4	8.149	12.409
2 Gy photon	CJ179	Fig 1b. from Riballo *et al* [32]	5	29.432	25.388
1.3 Gy photon	CJ179	Fig 1c. from Riballo *et al* [32]	3	147.481	70.994
Average				48.743	31.045
γ-H2AX for XLF-deficient cell lines				A	C
2 Gy photon	2BN	Fig 2a. from Beucher *et al* [50]	4	67.566	64.544

Table S3. Linear regression(Unrepaired DSBs=a*LET+b) through the data shown in Figure 5. R2is the coefficient of determination. The simulated results of ModelsAand Bare repeated 120 times.

	a	b	R^2
Model A	0.320±0.025	8.953±0.188	0.985
Model B	0.038±0.008	1.549±0.067	0.941
Chaudhary *et al* [52]	0.083±0.042	3.014±0.334	0.789

Table S4. Details for the experimental data used in this work.

**Group**	**Publication**	**IR**	**Cell**	**Statistics Type in literature**	**n****
Ku70/80	Andrin et al. Fig. 8 [69]	405 nm laser	U2OS	SEM*	30
	Uematsu et al. Fig. 2c [53]	365 nm laser	Xrs6	SD*	10
DNA-PKcs	Li et al. Fig. 2 [70]	365 nm laser	V3	SD	10
	Uematsu et al. Fig. 2 [53]	365 nm laser	V3	SD	10
	Mori et al. Fig. 3 [71]	365 nm laser	V3	SD	3
	Uematsu et al. Fig. 1d [53]	365 nm laser	V3	SD	10
CtIP	Qvist et al. Fig. 5d [42]	405 nm laser	U2OS	SD	12
EXO1	Bolderson et al. Fig. 1b [72]	365 nm laser	U2OS	SD	3
	Nishi et al. Fig. 6g [41]	405 nm laser	U2OS	SEM	3
Artemis	Gagne et al. Fig. 9c [43]	750 nm laser	HEK	SEM	8
Normal tissue	Fig 3c. from Wu *et al* [49]	4 Gy proton/7.5 MeV	NHLF	SEM	3
	Fig 2c. from Oeck *et al* [73]	3 Gy proton/32 MeV	MEF	SD	3
	Fig 2c from Oeck *et al* [73]	3 Gy proton/187 MeV	MEF	SD	3
	Fig 3b. from Wu *et al* [49]	2 Gy proton/7.5 MeV	NHLF	SEM	3
	Fig 3a. from Wu *et al* [49]	0.5 Gy proton/7.5 MeV	NHLF	SEM	3
	Fig 3c. from Wu *et al* [49]	4 Gy photon	NHLF	SEM	3
	Fig 3b. from Wu *et al* [49]	2 Gy photon	NHLF	SEM	3
	Fig 1b. from Riballo *et al* [32]	2 Gy photon	MCR-5	SEM	3
	Fig 1b. from Riballo *et al* [32]	2 Gy photon	HSF2	SEM	3
	Fig 2a. from Beucher *et al* [50]	2 Gy photon	C2906	SEM	3
	Fig 2b. from Beucher *et al* [50]	2 Gy photon	MEF	SEM	3
	Fig 2a. from Beucher *et al* [50]	1.3 Gy photon	1BR3	SEM	3
	Fig 3a. from Wu *et al* [49]	0.5Gy photon	NHLF	SEM	3
	Fig.5a from Ahmed *et al* [44]	0.5Gy photon	MEF	SD	3
	Fig.2b from Ahmed *et al* [44]	0.5Gy photon	Sertoli	SD	3
Artemis-deficient	Fig 2b. from Beucher *et al* [50]	2 Gy photon	MEF	SEM	3
	Fig 2a. from Beucher *et al* [50]	2 Gy photon	CJ179	SEM	3
	Fig 1b. from Riballo *et al* [32]	2 Gy photon	CJ179	SEM	3
	Fig 1c. from Riballo *et al* [32]	1.3 Gy photon	CJ179	SEM	3
XLF-deficient	Fig 2a. from Beucher *et al* [50]	2 Gy photon	2BN	SEM	3

*SEM is standard error of mean; SD is standard deviation; SEM = SD/(n)^0.5 .

** n is the number of independent measurements in the literature.
